# Network Structure Analysis Identifying Key Genes of Autism and Its Mechanism

**DOI:** 10.1155/2020/3753080

**Published:** 2020-03-23

**Authors:** Yanhui Wang, Yanming Kou, Dazhi Meng

**Affiliations:** ^1^College of Mathematics and Systems Science, Shandong University of Science and Technology, Qingdao 266590, China; ^2^College of Applied Science, Beijing University of Technology, Beijing 10024, China

## Abstract

Identifying the key genes of autism is of great significance for understanding its pathogenesis and improving the clinical level of medicine. In this paper, we use the structural parameters (average degree) of gene correlation networks to identify genes related to autism and study its pathogenesis. Based on the gene expression profiles of 82 autistic patients (the experimental group, *E*) and 64 healthy persons (the control group, C) in NCBI database, spearman correlation networks are established, and their average degrees under different thresholds are analyzed. It is found that average degrees of C and *E* are basically separable at the full thresholds. This indicates that there is a clear difference between the network structures of C and *E*, and it also suggests that this difference is related to the mechanism of disease. By annotating and enrichment analysis of the first 20 genes (MD-Gs) with significant difference in the average degree, we find that they are significantly related to gland development, cardiovascular development, and embryogenesis of nervous system, which support the results in Alter et al.'s original research. In addition, FIGF and CSF3 may play an important role in the mechanism of autism.

## 1. Introduction

Autism in children is a neurodevelopmental disorder characterized by different degrees of social interaction and communication disorders, narrow interests, repetitive stereotypes, and perceptual abnormalities. For quite a long time, autism was considered a rare disease with a prevalence of 2/10,000 to 4/10,000 [1]. In recent years, the global prevalence of autism has gradually increased. For example, in 2009, the prevalence was 1.57% in Britain and 1.64% in Japan and 2.64% in Korea in 2011. Biennial disease reports in the United States showed an upward trend, with 1.47% in 2014 [2]. Since 2000, surveys of autism prevalence in various provinces and municipalities in China have shown that the prevalence of autism ranges from 0.10% to 0.75% [3,4]. Some international organizations estimated that the global prevalence of autism was 1% [5]. Autism has undoubtedly become a serious global public health problem.

According to the current research, the hypothesis of the pathogenesis of autism mainly included immune dysfunction and synaptic dysfunction [6]. Hua et al. [7] believed that hundreds of pathogenic genes, susceptibility genes, and microRNAs were associated with autism, which clearly indicated that autism was a complex hereditary disease, and genetic variation/heritability was one of the main pathogenic factors. There are abundant evidences that genetic diseases are caused by complex interactions between multiple genes and some environmental risk factors [8,9]. Studies by Sarachana et al. [10] suggested that disorders in the expression of microRNAs may lead to autism. Kaushik et al. [11] pointed out that very low concentrations of psychoactive drugs altered the expression of key synaptic proteins in vitro, which may lead to neurological diseases such as autism by damaging neuronal development. Kawada et al. [12] showed that ER stress could induce abnormal maturation of cerebral cortical neurons in male ICR mouse embryos. Therefore, ER stress may be involved in the pathogenesis of autism. Lai et al. [13] pointed out that vitamin A (VA) is an essential nutrient for brain development. Because RAR signals inhibit the expression of CD38 in the hypothalamus of offspring, vitamin A deficiency during pregnancy may be one of the factors contributing to autism. Arioka et al. [14] pointed out that the repetition of the 15q11.2-q13.1 region was associated with mental disorders such as developmental retardation and autism. Most of the above results are from the perspective of single gene analysis of the cause of autism, but Hua et al. [7] believed that autism was a complex genetic disease, involving a large number of genes. The expression of multiple pathogenic genes should not be isolated, so in order to discover the pathogenic mechanism of autism, it is necessary not only to study single genes but also to study these pathogenic genes as a system.

With the development of genomics and high throughput technology, massive biological data are generated, such as protein structure and interaction data, genome expression profile data, gene expression regulation data, and metabolic data. Obviously, these data are the basis of systematic study of biology. Nanni et al. [15] made a statement that large genome-wide association studies (GWAS), Copy Number Variation (CNV) testing, and genome sequencing yielded many nonoverlapping genes, a fact that underlines the complex genetic heterogeneity of autism [16] and reflects the architecture of intracellular networks, in which several possible combinations of genetic variations are likely to lead to a common pathological phenotype [17,18]. So, network-based analysis will be helpful to study the pathogenesis of autism. Nanni et al. [15] pointed out that one of the challenges that network-based analyses face is the identification of the so-called disease modules, that is, gene networks associated with diseases [17]. Hu et al. [19] reported that stratifying the sample by cluster analyses revealed quantitative differences in gene expression which appear to correlate with severity of autism phenotype as well as gene expression profiles for each subtype which associate a “biological phenotype” (i.e., gene expression profile) to the respective functional/behavioral phenotype. The biological phenotypes reveal differences in some of the biological functions affecting individuals with autism, such as circadian rhythm dysregulation in the severe (*L*) phenotype, suggesting possible therapeutic interventions specific to this subgroup. On the other hand, overlapping genes among the phenotypes indicate dysregulation of genes controlling both neurological and metabolic functions that may lie at the core of autism [19]. Li et al. [20] integrated previously and newly generated data and developed a systems framework involving the interactome, gene expression, and genome sequencing to identify a protein interaction module with members strongly enriched for autism candidate genes. Hormozdiari et al. [21] developed a computational method, termed MAGI (merging affected genes into integrated networks), that simultaneously integrates protein-protein interactions and RNA-Seq expression profiles during brain development to discover “modules” enriched for de novo mutations in probands. They applied this method to recent exome sequencing of 1116 patients with autism and intellectual disability, discovering two distinct modules. The first module consists of 80 genes associated with Wnt, Notch, SWI/SNF, and NCOR complexes. The second module consists of 24 genes associated with synaptic function, including long-term potentiation and calcium signaling with higher levels of postnatal expression. Currently, the integrative analysis of multiple omics has emerged as an approach to provide a more comprehensive view of a disease. Nanni et al. [15] carried out a network-based meta-analysis of the genes reported as associated with autism by studies that involved genomics, epigenomics, and transcriptomics.

The structure of a network determines the function of the network system. Therefore, the study of the structure of networks plays an important role in understanding the function of biological networks. For the purpose of disease mechanism research, structural differences can be found through the structural comparison of disease and normal gene networks, which is likely to be an important cause of disease occurrence and development. A direct comparative analysis method of network structure is through the comparison of network structure parameters. Graph theory in mathematics has defined many network statistics, such as average degree, average kernel number, average path length, average point (edge) betweenness, module degree, and clustering coefficient. These statistics, also known as the structural parameters of the network, describe the structural characteristics of the network from an important point of view.

This paper constructs spearman correlation networks based on gene expression profile data in normal and autistic states, trying to reveal the structural differences between normal and autistic gene expression profile data sets by using network structure parameters. When the structural difference is significantly described, genes with maximally structural difference (MD-Gs) which contribute to this structural difference can be identified. Obviously, this structural difference is an important manifestation of the mechanism of organism transforming from normal to disease, and the MD-Gs causing structural difference are more likely to be the more direct cause of disease formation. Further analysis of biological functions of MD-Gs will help to explain the functional mechanism of disease occurrence.

## 2. Methods

### 2.1. Identifying of Autism-Related Genes

Nanni et al. [15] pointed out that while the analysis of epigenomics and transcriptomics from brain-derived samples can provide important insights into the potential mechanisms of disease etiology, there are relevant limitations with these types of studies (e.g., the quality of autopsy-derived tissue, sample size, influence of life experience, and cause of death) [22]. These barriers have been overcome by analyzing blood samples, and recent blood-based works have shown the usefulness of this alternative approach to gather insights into autism [23–25].

With that in mind, this study is based on the data of GSE25507 in NCBI on gene expression profiles of autistic patients and normal people. Gene expression microarrays covering greater than 47,000 unique RNA transcripts were done on RNA from peripheral blood lymphocytes (PBL) of children with autism (*n* = 82, *E*) and controls (*n* = 64, C). The data was pretreated with MAS5 and RMA. Each sample contains 23520 genes.

In fact, there may not be many genes closely related to autism, and the genes with different expression between C and *E* may be related to autism. Statistical analysis methods of hypothesis test can be used to screen the different genes between C and *E*. Let *X* and *Y* be samples of gene A in group I and group II, respectively. Through a series of hypothesis tests, we can judge whether there are differences in the expression of A between group I and group II. The outline of our method is as follows: we can first judge whether the distribution of *X* and *Y* is the same. The nonparametric test is better than the parametric test when there is no information about the population distribution. When the population distribution is known, it will become the disadvantage of nonparametric test without any prior knowledge. At this time, a nonparametric test method is not as accurate as that obtained by parametric test methods. Therefore, we first use KS test of two samples to preliminarily judge the consistency of distribution. If the distribution is not consistent, we think there is a difference between group I and group II; otherwise, we use KS test of single sample to check for normality. If both *X* and *Y* are normal, then *X* and *Y* are tested using *F* test to determine whether variance is homogeneous. If it is homogeneous with variance, *t*-test of two independent samples will be tested; otherwise, Welch's *t*-test of two independent samples will be used. If *X* and *Y* do not have normality, because the distribution of two-sample KS test is consistent, it is considered that they are consistent with the distribution shape, so the Mann–Whitney test can be further used. The cut-off thresholds of the FDR for the KS test of two samples, KS test of single sample, *F* test, and *t*-test (Welch's *t*-test, Mann–Whitney test) are 0.0005, 0.001, 0.001, and 0.001, respectively. 244 genes (called autism-related genes) expressed differently between C and *E* are screened as the research objects.

### 2.2. Constructing Spearman Correlation Networks

Spearman's rank correlation coefficient is a measure of the degree of dependence between two variables. It is used to measure the strength of the relationship between variables. The range of Spearman correlation coefficient is [−1, 1]. The greater the absolute value of Spearman correlation coefficient, the stronger the dependence between the two variables; on the contrary, the smaller the dependence.

Let *I* be a set of gene expression profiles. For any two genes *A*, *B* ∈ *I*, the Spearman correlation coefficient between them is recorded: *SCC*(*A*, *B*). Given the threshold *α* (0 < *α* ≤ 1), the Spearman correlation network under the threshold is constructed. The method is that each gene *A* ∈ *I* represents a node; if the absolute value of Spearman correlation coefficients of any two genes *A*, *B* ∈ *I* is such that |*SCC*(*A*, *B*)| ≥ *α* there is a connection between nodes *A* and *B*; otherwise there is no connection. In this way, a Spearman correlation network of gene expression profiles under a threshold is established.

### 2.3. The Maximally Structural Difference Genes

Current studies suggest that autism may be caused by many genetic factors. There are so many genes related to autism, which all play a role in the mechanism of autism. In the analysis of network structure parameters, the genes that contribute a lot to parameter difference are considered as key genes. As the number of key genes is related to the complexity of gene annotation analysis, and our aim is to check whether the analysis method using network structure parameters is effective to study disease mechanisms, 244 genes are sequenced according to the degree difference between C and *E* under the full threshold and then the top 20 genes are selected for analysis. These 20 genes are called the maximally structural difference genes (MD-Gs). The function of a gene is determined by the interaction between genes. This means that the degrees of genes in networks reflect the functional difference. The larger the degree difference is, the greater the variation of the gene quantity associated with the gene in the network is and the more the function of the gene increases or decreases in the network. It is better to measure using the absolute value of the degree difference between C and *E* rather than relative changes of the degree, (|*E* − *C*|/*C*), as the relative change of the degree depends on C. The selection method is as follows: firstly, the degree of each node in the correlation network between C and *E* under 0.1–0.9 thresholds is calculated, respectively; secondly, the absolute value of the degree difference between C and *E* is calculated for 244 autism-related genes under each threshold, and then the average of the absolute values of the degree difference of each gene between C and *E* is ranked from large to small.

### 2.4. Enrichment Analysis of MD-Gs

The express analysis tool of Metascape (http://metascape.org) [26] is used to analyze the metabolic pathways and processes of MD-Gs. For each given gene list, pathway and process enrichment analysis has been carried out with the following ontology sources: KEGG Pathway, GO Biological Processes, Reactome Gene Sets, Canonical Pathways, and CORUM. All genes in the genome have been used as the enrichment background. Terms with a *p* value <0.01, a minimum count of 3, and an enrichment factor >1.5 (the enrichment factor is the ratio between the observed counts and the counts expected by chance) are collected and grouped into clusters based on their membership similarities. More specifically, *p* values are calculated based on the accumulative hypergeometric distribution [27], and q-values are calculated using the Benjamini–Hochberg procedure to account for multiple testings [28]. Kappa scores [29] are used as the similarity metric when performing hierarchical clustering on the enriched terms, and subtrees with a similarity of >0.3 are considered a cluster. The most statistically significant term within a cluster is chosen to represent the cluster.

## 3. Results

In the network, the degree of a node can be used to indicate its importance. The greater the degree of a node is, the more important the node is in the network [30]. The average degree of a Spearman correlation network is calculated under different thresholds (*α*  ∈ {0.1, 0.2,…, 0.9}) for C and *E*, as shown in Table 1.

It can be seen from Table 1 that the average degree of Spearman correlation network in C is higher than that in *E* under the full threshold (0.1–0.9). According to the conclusion of the numerical experiment, the probability that the average degrees of random networks are separable among the threshold (0.1–0.9) is about 20*%*, and so the confidence in the fact that the average degrees of Spearman correlation networks of C and *E* are separable among the threshold (0.1–0.9) is about 80*%*. This indicates that the correlation networks of genes between C and *E* have some structural differences. This may be due to the different degree of nodes between C and *E*. The difference in the degree of some genes between C and *E* indicates that the association of these genes with other nodes in the network changes leads to the difference of network structure between C and *E*. The structure determines the function. These genes that lead to structural differences are likely to be closely related to autism.

MD-Gs are shown in Table 2.

The top six clusters of functions related to MD-Gs are shown in Table 3.

## 4. Discussion

### 4.1. Annotation Analysis of Single MD-G

Referring to the literature, OMIM, KEGG, and NCBI databases, the functions of OGFRP1 and Loc284788 are not clear in Table 2; annotations of the other 18 genes are detailed in the Supplementary Materials ([Supplementary-material supplementary-material-1]).

Data shows that MED13, MED12, CDK8, and Cycn C (CycC) are the main entrances to carcinogenic and developmental signal/gene expression [31]. Mutations in MED13 cause autism [9].

ARHGDIG is also known as RhoGDI3. RhoGDI3 may induce the downregulation of RhoG and RhoB [32]. Neurotrophic factors are involved in neurodevelopment, neuronal survival, and synaptogenesis and are considered to be important substances affecting autism [33]. ARHGIG is involved in hsa04722 (neurotrophic factor signaling pathway), so ARHGIG may affect autism through neurotrophic factor signaling pathway.

It was found that NDRG4−/− mice were born at the expected Mendelian rate and looked normal and fertile. However, NDRG4−/− mice had deficiencies in spatial learning and memory and showed increased sensitivity to ischemic stress after middle cerebral artery occlusion. Consistent with these findings, the expression of neuroprotective factor BDNF in NDRG4−/− mice decreased [34]. Reference [35] demonstrated for the first time that NDRG4 may be a potential tumor suppressor gene and prognostic marker for gastric cancer. Chen et al. [36] explained the risk of gastric cancer caused by hypermethylation of NDRG4 promoter. Qu et al. [37] illustrated the potential role of NDRG4 in intestinal development, nervous system, and immune system. Sarachana et al. [10] revealed that there is a link between autism and gastrointestinal diseases, and there is also a link between genes involved in gastrointestinal diseases and the occurrence of autism. Therefore, NDRG4 may affect the development of nervous system on the one hand and gastrointestinal function on the other hand, which is closely related to the generation of autism.

POU3F2 is located downstream of SIM1 and controls the expression of oxytocin in the preoptic area of hypothalamic neuroendocrine [38]. This gene encodes a member of POU-III neurotranscription factor and plays an important role in brain development. Lin et al. [39] demonstrated that POU3F2 plays a role in neuronal differentiation. Hashizume et al. [40] showed that POU3F2 is related to cognitive function and adult hippocampal neurogenesis. Belinson et al. [41] demonstrated that transcriptional disorders of POU3F2/BRN-2 in the embryonic brain can lead to autism.

SHANK mutations account for ∼1*%* of patients with autism and were detected in the whole spectrum of autism with a gradient of severity in cognitive impairment: mutations in SHANK1 were rare (0.04*%*) and present in males with normal IQ and autism; mutations in SHANK2 were present in 0.17*%* of patients with autism and mild intellectual disability; mutations in SHANK3 were present in 0.69*%* of patients with autism and up to 2.12*%* of the cases with moderate to profound intellectual disability [42]. Mutations in SHANK3 or changes in protein levels are associated with neurodevelopmental disorders, such as Phelan-McDermid syndrome, autism, and schizophrenia [43]. Campbell and Sheng [44] identified USP8/UBPY as a deubiquitinase that regulates the ubiquitination and protein levels of Shank3 and Shank1. Therefore, USP8 and SHANK3 synergistically affect the development of autism.

Depending on the function of brain regions, the candidate genes for autism affect synaptic transmission through three pathways: nerve regeneration, cell adhesion, and ion channel activity [6]. FIGF, also known as VEGFD, has hsa0451: focal adhesion and hsa04151: PI3K-Akt signaling pathway. PI3K transfers a phosphoric acid group to site 3. The products formed have important effects on cell function. For example, PIP2 converts to PI-3,4,5-triphosphate, which can regulate cell adhesion, growth, and survival. This suggests that FIGF may influence the mechanism of autism through cell adhesion pathway. In addition, FIGF has hsa04014 (Ras signaling pathway), hsa04015 (Rap1 signaling pathway), hsa04151 (PI3K-Akt signaling pathway), and hsa04010 (MAPK signaling pathway). In mammals, an hsa04010 signal transduction pathway (ERK1/2 signal transduction pathway) regulates cell growth and differentiation, and it cooperates with hsa04014 and hsa04015 to influence the development of nervous system. Rap belongs to the Ras family and contains Rap1 and Rap2 subclasses. Rap control signaling pathways in cells are closely related to the formation of cell polarity, cell proliferation, differentiation and carcinogenesis, cell adhesion, and movement and further affect some important physiological functions at the level of tissues and organs, such as the establishment of nerve polarity, synaptic growth, synaptic plasticity, and neuron migration [45]. The Raf/ERK overexpression cell model established by Yin [6] has decreased migration ability, disturbance of excitatory synapse formation, and maturation of dendritic spines, suggesting that the upregulation of Raf/ERK expression may affect the development of neurons and lead to nervous system imbalance. In addition, studies have found that deletion of chromosome 16 is associated with autism, and the MAPK3 gene encoding ERKl protein is located on chromosome 16 [1]. Erk2 deficiency in 22q11.2 was also associated with the onset of autism [46,47]. The MAPK gene is located in the 22q1.3 DGS region, which is deleted in many patients, leading to a series of heart, skin, and nervous system abnormalities [46–49]. Metabolic pathway hsa04151 (PI3K-Akt signaling pathway) widely exists in various nerve cells. It is an important pathway for membrane receptor signal transduction into cells. It has cell biological functions such as regulating cell proliferation, differentiation, metabolism, and antiapoptosis. Abnormal expression of PI3K-Akt signaling pathway can lead to symptomatic autism [50,51]. Ras, PI3K, and Rap1 are involved in the MAPK signaling pathway (https://www.biomart.cn/news/10/119965.htm). This suggests that signal transduction pathways hsa04014, hsa04015, and hsa04151 are interrelated through hsa04010. However, how they interact to produce autism remains unclear.

According to studies, the hypothesis of the pathogenesis of autism mainly includes immune dysfunction and synaptic dysfunction [6]. The metabolic function of CSF3 is related to immunity or infectious diseases, and hsa04657 (IL-17 signaling pathway) is related to IL-17. Choi et al. [52] pointed out that immune cells activated in maternal inflammation produce an effector molecule (IL-17), interfering with fetal brain development, leading to autism, and blocking this signal can restore normal behavior and brain structure. Abnormal expression of hsa04151 signaling pathway can lead to autism [50,51]. This suggests that CSF3 may influence the mechanism of autism through the immune system.

From the single gene annotation analysis, it can be seen that the abnormalities of MED13 [9], POU3F2 [41], and USP8 [44] have been confirmed to induce autism. FIGF regulates the signal transduction pathways hsa04014, hsa04015, hsa04151, and hsa04010. If there is a problem in one of them, it may cause the development disorder of nervous system and then lead to the occurrence of autism. However, the mechanism of how they interact to produce autism needs further study. This suggests that FIGF plays an important role in the mechanism of autism. CSF3 is involved in hsa04060, hsa04151, hsa04630, hsa04640, and hsa04657. The abnormalities of hsa04657 [52] and hsa04151 [50,51] have been confirmed to induce autism. This suggests that CSF3 is related to autism through the immune system. On the one hand, NDRG4 may affect the development of nervous system; on the other hand, it may affect the gastrointestinal function, which is closely related to the occurrence of autism.

Gene annotation shows that NDRG4, JUP, and PML are all related to gastrointestinal function. Enrichment analysis shows that vascular development and embryogenesis are significant. These conclusions support the viewpoint of Alter et al. [53]; that is, global levels of gene expression regulation may impact systems other than the brain.

### 4.2. Enrichment Analysis

#### 4.2.1. P1

HOXD9 has GO:0048935 (peripheral nervous system neuron development) and GO:0048934 (peripheral nervous system neuron differentiation). PML has GO:0006977, that is, DNA damage response and signal transduction by p53 class mediator resulting in cell cycle arrest. POU3F2 has GO:0021985 (neurohypophysis development) and MESP1 has GO:0042664 (negative regulation of endodermal cell fate specification). It can be seen that P1 is related to the development of nervous system glands, which indicates that the research on the development of nervous system glands is helpful to study the pathogenesis of autism.

#### 4.2.2. P2

FSD1 has GO:0060236 (regulation of mitotic spindle organization) and GO:0031122 (cytoplasmic microtubule organization); POU3F2 has GO:0021979 (hypothalamus cell differentiation); FIGF has GO:0060754 (positive regulation of mast cell chemotaxis), GO:0060753 (regulation of mast cell chemotaxis), and GO:0050930 (induction of positive chemotaxis); USP8 has GO:0071549 (cellular response to dexamethasone stimulus) and GO:0099170 (postsynaptic modulation of chemical synaptic transmission). Mast cells are related to immunity. Dexamethasone is related to endocrine system. It has anti-inflammatory, antiendotoxin, immunosuppressive, antishock, and enhanced stress response. This suggests that the inflammation caused by mast cells in the blood affects the differentiation of hypothalamic cells through the endocrine system and then is related to autism.

#### 4.2.3. P3

JUP has GO:0086073 (bundle of His cell-Purkinje myocyte adhesion involved in cell communication), GO:0098911 (regulation of ventricular cardiac muscle cell action potential), and GO:0086069 (bundle of His cell to Purkinje myocyte communication); MESP1 has GO:0090082 (positive regulation of heart induction by negative regulation of canonical Wnt signaling pathway) and GO:0090081 (regulation of heart induction by regulation of canonical Wnt signaling pathway). This suggests that the positive regulation of Wnt signaling pathway on the induction of cardiac cells, especially Purkinje cells, is significantly related to the mechanism of autism.

#### 4.2.4. P4

HOXD9 has GO:0035115 (embryonic forelimb morphogenesis); MESP1 has GO:0042664 (negative regulation of endodermal cell fate specification); NDRG4 has GO:0010642 (negative regulation of platelet-derived growth factor receptor signaling pathway). PDGF is an important mitogenic agent, which can stimulate the division and proliferation of vascular smooth muscle cells, fibroblasts, glial cells, and other cells and regulate the individual development and cell differentiation. This suggests that fetal dysplasia may induce autism.

#### 4.2.5. P5

FIGF has GO:0060754, GO:0060753, and GO:0050930, which are all related to the regulation of mast cell chemotaxis. JUP has GO:0086073, GO:0098911, and GO:0086069, which are related to Purkinje myocytes in cardiomyocytes. PML has GO:1902187 (negative regulation of virtual release from host cell), GO:0006977 (DNA damage response, signal transmission by p53 class mediator results in cell cycle arrest), and GO:0050713 (negative regulation of interleukin-1 beta secret), which are related to immunity. MESP1 has GO:0090082 and GO:0090081, which are the regulation of Wnt signaling pathway on cardiac induction. This suggests that the effect of inflammation on vascular development contains information on the pathogenesis of autism.

#### 4.2.6. P6

CSF3 has GO:0014068 (positive regulation of phosphatidylinositol 3-kinase signaling). Abnormal expression of hsa04151 signaling pathway may lead to symptomatic autism (Bill and Geschwind [50] and Hu [19]). This suggests that hsa04151 may induce autism symptoms through P6. MED13 has GO:0070328 (triglyceride homeostasis), GO:0055090 (acylglycerol homeostasis), and GO:0042632 (cholesterol homeostasis). MED13 is related to development [31], and its mutation can cause autism [9]. This indicates that MED13 affects development through P6 and then induces autism.

According to gene enrichment analysis, the functions of MD-Gs are mainly reflected in six aspects: gland development, cell division, Wnt signal pathway regulation, embryogenesis, vascular development, and DNA binding transcription factor activity regulation. According to the GO function analysis of the involved genes, these six aspects can be summarized as follows: (1) gland development of the nervous system; (2) inflammation caused by mast cells in the blood affecting the differentiation of hypothalamic cells through the endocrine system; (3) Wnt signal pathway regulating the positive regulation of platelet-derived growth factor receptor induced by cardiac cells, especially Purkinje cells; (4) platelet-derived growth factor receptor signaling pathway regulating embryogenesis; (5) the regulation of inflammatory immunity on vascular development; and (6) hsa04151 signaling pathway containing CSF3 and MED13 affecting the mechanism of autism through the regulation of DNA binding transcription factor activity.

The results of gene annotation and enrichment analysis support the research of Alter et al. [53] on the relationship between father's age, neurodevelopmental disorder, and immune function. Children with autism have immunological abnormalities and it has been previously reported that gene expression differences were found in immune cells of children with autism [54]. A relationship with paternal age suggests that factors influencing global levels of gene expression regulation may be transmitted across the germ line. Additionally, findings in the blood suggest the possibility for effects of paternal age on immune function. Given the links between paternal age, neurodevelopomental disorders, and immune function, it seems that paternal age might also have a more generalized effect on immune function as it was found to have on neurodevelopment [53].

### 4.3. Functional Difference Analysis of MD-Gs between C and E

**Figure 1:**
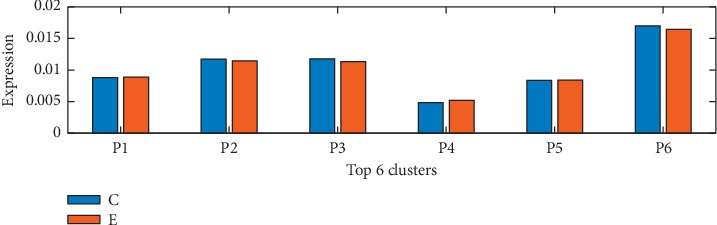
Expression levels of P1–P6.

To further analyze the differences of P1–P6 between C and *E*, the expression levels of the function in C and *E* are calculated as follows. Firstly, the average expression levels of MD-Gs in C and *E* are calculated, respectively. If *A*_1_, *A*_2_,…, *A*_*k*_ have function F, the sum of the average expression levels of *A*_1_, *A*_2_,…, *A*_*k*_ in C is the total expression level of function F in C. Similarly, the total expression level of function F in *E* could be calculated; see Figure 1.

It is found that the maximum of the average expression difference of gene between C and *E* is 0.042, the minimum is 2.1632e-09, the average value is 4.3746e-04, and the variance is 2.5730e-06. This shows that there is no significant difference between gene expression profiles of C and *E*. In Figure 1, only the difference between C and *E* in P6 is 5.6204e-04 larger than the average. This suggests that the expression of P6 in autism is lower than that in healthy people. In addition, although the difference of expression of C and *E* on P1–P5 is lower than the average, it cannot be denied that the slight difference may contain the pathogenic mechanism information of autism.

According to the analysis of the difference of gene function expression, people with autism have lower expression in the regulation of DNA binding transcription factor activity than healthy people, which suggests that autism has defects in the regulation of DNA binding transcription factor activity.

## 5. Conclusion

The topological structure of networks plays an important role in understanding the function of biological networks. The analysis method of network structure parameters is used to study the pathogenesis of autism from the genetic level. Spearman correlation networks are established by using gene expression profiles of C and *E*, and their average degrees are analyzed. It is found that the average degrees of C and *E* are separable under the full threshold with confidence of 80*%*. This shows that there are obvious structural differences between the gene networks of C and *E*, which leads to functional differences. The first 20 genes with significant difference in average degree are selected for enrichment analysis. It is found that they are significantly related to gland development, cardiovascular development, and embryonic organ morphogenesis of the nervous system. This does not only support the view in Alter et al.'s work [53], that is, altered regulation of transcription may underlie decreased variance and may increase risk for autism, but also supports the conclusion that developmental disorders of the nervous system [33,55] may cause symptoms of autism. However, the mechanism of cardiovascular development on autism is rarely reported. The mechanism of how the development of nervous system glands, cardiovascular system, and embryonic organ morphogenesis induces autism needs further study. In addition, PI3K-Akt signal pathway and RAS-MAPK signal pathway are clearly related to autism, and the abnormal expression of gene CSF3 and FIGF may cause the abnormal expression of these signal pathways. This provides a theoretical basis for further medical experiments to study the mechanism of autism. These results show that the network structure parameter analysis method is an effective method.

## Figures and Tables

**Table 1:** The average degree.

Thresholds	0.1	0.2	0.3	0.4	0.5	0.6	0.7	0.8	0.9
C	173.82	113.57	69.93	38.79	17.09	5.28	1.09	0.26	0.03
E	170.7	110.09	65.55	33.61	13.79	4.11	0.86	0.07	0.02

**Table 2:** MD-Gs.

No	Genes	Description	Expression
1	HYI	Hydroxypyruvate isomerase	*E* > *C*
2	PPP1R37	Protein phosphatase 1 regulatory subunit 37	*E* > *C*
3	HOXD9	Homeobox D9	*E* > *C*
4	EXTL1	Exostosin like glycosyltransferase 1	*E* > *C*
5	MED13	Mediator complex subunit 13	*C* > *E*
6	C7orf63	Cilia and flagella associated protein 69	*E* > *C*
7	ARHGDIG	Rho GDP dissociation inhibitor gamma	*E* > *C*
8	JUP/KRT17	Junction plakoglobin/type I keratin, acidic	*E* > *C*
9	NPY4R	Neuropeptide Y receptor Y4-2	*E* > *C*
10	NDRG4	NDRG family member 4	*E* > *C*
11	POU3F2	POU class 3 homeobox 2	*E* > *C*
12	LOC284788	Uncharacterized LOC284788	*E* > *C*
13	USP8	Ubiquitin specific peptidase 8	*C* > *E*
14	OGFRP1	Opioid growth factor receptor pseudogene 1	*E* > *C*
15	FIGF	Vascular endothelial growth factor D	*E* > *C*
16	ZBP1	Insulin like growth factor 2 mRNA binding protein 1	*C* > *E*
17	PML	Promyelocytic leukemia	*C* > *E*
18	CSF3	Colony stimulating factor 3	*E* > *C*
19	FSD1	Fibronectin type III and SPRY domain containing 1	*E* > *C*
20	MESP1	Mesoderm posterior bHLH transcription factor 1	*E* > *C*

The column of “Expression” shows the comparison of the average expression profiles of key structural genes between C and *E*.

**Table 3:** Top 6 clusters with their representative enriched terms (one per cluster).

No	GO	Description	Count	%	Log 10 (P)	Log 10 (q)	Genes
P1	GO:0048732	Gland development	4	20	−3.40	0.00	HOXD9, PML, POU3F2, MESP1
P2	GO:0051301	Cell division	4	20	−2.88	0.00	FIGF, USP8, FSD1, POU3F2
P3	GO:G0030111	Regulation of Wnt signaling pathway	3	15	−2.79	0.00	JUP, USP8, MESP1
P4	GO:0048562	Embryonic organ morphogenesis	3	15	−2.78	0.00	HOXD9, MESP1, NDRG4
P5	GO:0001568	Blood vessel development	4	20	−2.5	0.00	FIGF, JUP, PML, MESP1
P6	GO:0051090	Regulation of DNA binding transcription factor activity	3	15	−2.29	0.00	CSF3, JUP, CMED13

“Count” is the number of genes in the user-provided lists with membership in the given ontology term. “%” is the percentage of all of the user-provided genes that are found in the given ontology term (only input genes with at least one ontology term annotation are included in the calculation). “Log10 (P)” is the *p* value in log base 10. “Log10 (q)” is the multitest adjusted *p* value in log base 10. The category of clusters in Table 3 is GO Biological Processes.

## Data Availability

The processed data used to support the findings of this study are included within the supplementary information files).

## Supplementary Materials

Supplementary Materials Data1: sheet1 is about 244 genes related to ASD; sheet2 is about the expression profile of 244 genes related to ASD in the control group (C); sheet3 is about the expression profile of 244 genes related to ASD in the experimental group (*E*). Data2: sheet1 and sheet2 are the Spearman correlation coefficients of C and *E*, respectively. Data3: the Spearman correlation networks of C at different threshold (0.1–1) (sheet1–sheet10). Data4: the Spearman correlation networks of *E* at different threshold (0.1–1) (sheet1–sheet10). Data5: comparison of node degree of C and *E* 's correlation networks under different thresholds (sheet1–sheet4). Data6: analysis of structural key genes (sheet1–sheet5).
