# Supplementary material for: Network Structure Analysis Identifying Key Genes of Autism and Its Mechanism
Source: Comput Math Methods Med. 2020 Mar 23;2020:3753080. doi: 10.1155/2020/3753080 (PMC7125446; doi:10.1155/2020/3753080)
Supplement: Supplementary Materials — Data1: sheet1 is about 244 genes related to ASD; sheet2 is about the expression profile of 244 genes related to ASD in the control group (C); sheet3 is about the expression profile of 244 genes related to ASD in the experimental group (E). Data2: sheet1 and sheet2 are the Spearman correlation coefficients of C and E, respectively. Data3: the Spearman correlation networks of C at different threshold (0.1–1) (sheet1–sheet10). Data4: the Spearman correlation networks of E at different threshold (0.1–1) (sheet1–sheet10). Data5: comparison of node degree of C and E 's correlation networks under different thresholds (sheet1–sheet4). Data6: analysis of structural key genes (sheet1–sheet5). [file 3753080.f1.zip › 3753080.f1/mat.3753080.v1-1.docx]

**Appendix: Annotation of Structural Key Genes**

Using bioinformatics databases KEGG, GO and OMIM, as well as literature to mine the relevant information of single structural key genes, except that OGFRP1 and LOC284788 are genes not yet described, the annotations of other genes are as follows.

**HYI** encodes a hypothetical hydroxypyruvate isomerase, which may catalyze the conversion of hydroxypyruvate to 2-hydroxy-3-oxopropionic acid and may participate in the transport and metabolism of carbohydrates. EXTL1 is a tumor suppressor gene. EXT1 and EXT2 are associated with hereditary multiple exostosis and encode bifunctional glycosyltransferases (provided by RefSeq, Jul 2008) necessary for chain polymerization of heparin sulfate and its analogue heparin. 5-hydroxytryptamine (5-HT) is one of the earliest neurotransmitters in brain development. The disorder of 5-HT system is closely related to the occurrence and development of autism (Lei et al. 2018). Gene TPH2 defect can affect the formation of 5-HT neuronal circuits, so autism is closely related to THP2 (Lei et al. 2018). The genes TPH2, HYI and EXTL1 participate in the metabolic pathway hsa01100, so the genes HYI and EXTL1 may affect autism through the metabolic pathway hsa01100.

**HOXD9** belongs to the homeobox gene family. Homeobox genes encode highly conserved family of transcription factors, which play an important role in the morphogenesis of all multicellular organisms. Mammals have four similar homologous box gene clusters, HOXA, HOXB, HOXC and HOXD, which are located on different chromosomes and arranged in series by 9 to 11 genes. HOXD9 is one of several homologous box HOXD genes located on chromosome 2q31-2q37. The exact role of this gene has not been determined (provided by RefSeq, Jul 2008).

The protein encoded by **MED13** is a subunit of a macromolecule-mediated complex with DNA-binding transcription factor and RNA polymerase II (Sato et al. 2004). Data show that MED13, MED12, CDK8 and Cycn C (CycC) are the main entrances to carcinogenic and developmental signal/gene expression (Clark et al. 2015). Gene MED13 encodes the components of the mediator complex (also known as TRAP, SMCC, DRIP or ARC), which is considered to be the transcriptional co-activator complex necessary for almost all gene expression. Mediator complexes are recruited by transcriptional activators or nuclear receptors to induce gene expression, possibly by interacting with RNA polymerase II and promoting the formation of pre-transcriptional priming complexes. The product of the gene forms a subcomplex with MED12, cyclin C and C DK8, which can be negatively activated by mediators (provided by RefSeq, Jul 2008). Mutations in the MED13 gene cause autism (Pinero et al. 2015).

**ARHGDIG** is also known as RhoGDI3. RhoGDI3 may induce the downregulation of RhoG and RhoB (de Leon-Bautista et al. 2016). Neurotrophic factors are involved in neurodevelopment, neuronal survival and synaptogenesis, and are considered to be important substances affecting autism (Toma et al. 2013). ARHGIG is involved in the metabolic pathway hsa04722 (neurotrophic factor signaling pathway), so ARHGIG may affect autism through neurotrophic factor signaling pathway.

**JUP/KRT17** encodes a major cytoplasmic protein, which is the only known common component of desmosomes and subumbilical plaques at the junction. This protein forms a unique complex with cadherin and desmosome cadherin and is a member of the catenin family (provided by RefSeq, Jul 2008). JUP interacts with p53R175H to reduce the level of beta-catenin (Alaee et al., 2018). This literature has not been found. Sarachana et al (2010) reveals that there is a link between autism and gastrointestinal diseases, and that genes involved in gastrointestinal diseases are also associated with the occurrence of autism. Gene JUP plays an important role in the metabolic pathway hsa05226 (gastric cancer metabolic pathway), so gene JUP may also be one of the important genes affecting autism.

The protein encoded by **PML** gene is a member of TRIM family. This phosphoprotein is located in the nucleosome, where it acts as a transcription factor and a tumor suppressor. Its expression is related to cell cycle and regulates the response of p53 to carcinogenic signals (provided by RefSeq, Jul 2008). The gene and JUP participate in cancer-related metabolic pathways hsa05200, hsa05202 and hsa05221. Combined with the above JUP annotations, it is possible that PML is also associated with autism by affecting gastrointestinal function.

**NPY4R** is also known as PP1. The inhibition of JNK-c-Jun signal transduction by pancreatic polypeptide receptor 1 (PP1) is one of the important anti-tumor mechanisms of neuron membrane cytoskeleton protein 4.1 (4.1N) (Wang et al. 2016). Warrieret al. (2013) confirmed that GABRB3 is one of the important genes affecting autism. GABRB3 and NPY4R are involved in the metabolic pathway hsa04080 (neuroactive ligand-receptor interaction), so NPY4R may be closely related to autism by affecting neuronal development.

**PPP1R37** is known by KEGG that PPP1R37 interacts with PP1 in [BR: ko01009]. Therefore, the annotation of PP1 (NPY4R) indicated that PPP1R37 may affect the development of neurons.

**NDRG4**, also known as SMAP8, is most expressed in adult and fetal brains, and in the amygdala, cerebellum and thalamus (Hirosawa et al. 1999). This gene is a member of the N-myc down-regulated gene. It belongs to the alpha/beta hydrolase superfamily. The protein encoded by this gene is a cytoplasmic protein necessary for the progression and survival of primary astrocytes, and may be involved in the regulation of mitotic signal transduction in vascular smooth muscle cells. Selective splicing results in multiple transcripts encoding different isomers (provided by RefSeq, Jun 2011). Yamamoto et al. (2011) It was found that Ndrg4-/- mice were born at the expected Mendelian rate and looked normal and fertile. However, Ndrg4-/- mice had deficiencies in spatial learning and memory, and showed increased sensitivity to ischemic stress after middle cerebral artery occlusion. Consistent with these findings, the expression of neuroprotective factor Bdnf in Ndrg4-/- mice decreased. Zhang et al. (2018) demonstrated for the first time that NDRG4 may be a potential tumor suppressor gene and prognostic marker for gastric cancer. Chen et al. (2017) explained the risk of gastric cancer caused by hypermethylation of NDRG4 promoter. Qu et al. (2016) illustrates the potential role of NDRG4 in intestinal development, nervous system and immune system. Sarachana et al. (2010) revealed that there is a link between autism and gastrointestinal diseases, and there is also a link between genes involved in gastrointestinal diseases and the occurrence of autism. Therefore, NDRG4 gene may affect the development of nervous system on the one hand, and gastrointestinal function on the other hand, which is closely related to the generation of autism.

**POU3F2** belongs to a large family of transcription factors that bind to the octamer DNA sequence ATGCAAAT. Most of these proteins share a highly homologous region called POU domain, which occurs in several mammalian transcription factors, including octamer binding protein Oct1 (POU2F1) and Oct2 (POU2F2) and pituitary protein Pit1 (PIT1). Class III POU gene is mainly expressed in the central nervous system (CNS). CNS-specific transcription factors may play an important role in mammalian neurogenesis by regulating their different gene expression patterns (Schreiber et al. 1993; Atanasoski et al. 1995). The protein is expressed in CNS of the developing and adult brain. POU transcription factor B is involved in the development of Schwann cells. Jaegle et al. (2003) suggested that Brn2 and Oct6 play a role together as active regulators of Schwann cell development. POU3F2 is located downstream of SIM1 and controls the expression of oxytocin in the preoptic area of hypothalamic neuroendocrine (Kasher et al. 2016). This gene encodes a member of POU-III neurotranscription factor and plays an important role in brain development. Lin et al. (2018) demonstrated that POU3F2 plays a role in neuronal differentiation. Hashizume et al. (2017) shows that POU3F2 is related to cognitive function and adult hippocampal neurogenesis. Belinson et al. (2016) demonstrated that transcriptional disorders of POU3F2/BRN-2 in the embryonic brain can lead to autism.

The protein encoded by USP8 is a member of the bispecific protein phosphatase subfamily. These phosphatases inactivate their target kinases by dephosphorylating phosphoserine/threonine and phosphotyrosine residues. They negatively regulate the members of the mitogen-activated protein (MAP) kinase superfamily (MAPK/ERK, SAPK/JNK, p38), which is related to cell proliferation and differentiation. Different members of bispecific phosphatase family show different substrate specificity for different MAP kinases, different tissue distribution and subcellular localization, and different expression patterns induced by extracellular stimulation. The gene product inactivates SAPK/JNK and p38, mainly expressed in adult brain, heart and skeletal muscle, localized in cytoplasm, and induced by nerve growth factor and insulin. There is an intron-free pseudogene of DUSP8 on chromosome 10q11.2 (provided by RefSeq, Jul 2008). Berlin et al. (2010) concluded that USP8-STAM complex is a protective mechanism regulating the early endosomal sorting of EGFR between lysosomal degradation and recycling pathways. Mutations in SHANK3 or changes in protein levels are associated with neurodevelopmental disorders, such as Phelan-McDermid syndrome, autism spectrum disorders and schizophrenia (Audrey et al. 2014). Kerrisk et al. (2018) identified USP8/UBPY as a deubiquitinase that regulates the ubiquitination and protein levels of Shank3 and Shank1. Therefore, USP8 and SHANK3 synergistically affect the development of autism.

FIGF is also known as VEGFD. Vascular endothelial growth factor (VEGF) is a regulator of endothelial cell growth and function. It is most expressed in lung, heart, small intestine and fetal lung, but less expressed in skeletal muscle, colon and pancreas (Yamada et al. 1997). The protein encoded by this gene is a member of the platelet-derived growth factor/vascular endothelial growth factor (PDGF/VEGF) family, which is active in angiogenesis, lymphangiogenesis and endothelial cell growth. This secreted protein undergoes complex protein hydrolysis maturation, produces a variety of processing forms, binding and activating the receptors of VEGFR-2 and VEGFR-3. This protein is similar in structure and function to vascular endothelial growth factor C. Read-out transcription (provided by RefSeq, Feb 2011) was observed between this site and the upstream PIR (GeneID 8544) site. FIGF is involved in Ras signaling pathway, which may be involved in the pathogenesis of autism (Yin et al. 2012). Therefore, the gene FIGF may affect autism through Ras signaling pathway.

**CSF3**, a protein encoded by this gene, is a cytokine that controls the production, differentiation and function of granulocytes. Active proteins exist outside cells (provided by RefSeq, May 2010). Choi et al. (2016) points out that immune cells activated in maternal inflammation produce an effector molecule (IL-17), which interferes with fetal brain development and leads to autism. Blocking this signal can restore normal behavior and brain structure. Therefore, metabolic pathway hsa04657 (IL-17 signaling pathway) is an important factor affecting autism. As a gene controlling metabolic pathway hsa04657, CSF3 is likely to affect the development of autism through metabolic pathway hsa04657. PI3K-Akt signaling pathway widely exists in various nerve cells. It is an important pathway of membrane receptor signal transduction into cells. It has cell biological functions such as regulating cell proliferation, differentiation, metabolism and anti-apoptosis. Abnormal expression of PI3K-Akt signaling pathway can lead to symptomatic autism (Bill 2009; Hu et al. 2012). FIGF and CSF3 are involved in PI3K-Akt signaling pathway. Therefore, the genes FIGF and CSF3 may be associated with autism.

**ZBP1**, also known as DLM1 or DAI, is a DNA-dependent activator of interferon regulatory factors. It is a DNA sensor that can activate type I interferon and other immune responses. In addition, interferon-induced artificial expression of DAI in mouse fibroblasts selectively enhanced induction of DNA-mediated interferon I and other genes involved in innate immunity. On the other hand, DNA interference of DAI mRNA inhibits the induction of DAI gene under DNA stimulation. In addition, DAI binds to double-stranded DNA, which enhances its association with IRF3 transcription factors and TBK1 serine/threonine kinase. These observations highlight the overall role of DAI in the activation of DNA-mediated innate immune responses and provide new insights into the signaling mechanisms of DNA-related antimicrobial immunity and autoimmune disorders (Takaoka et al. 2007).

**FSD1** is abundant only in the brain (Carim-Todd et al. 2001). Manabe et al. (2002) It was found that endogenous mammal Fsd1 was associated with microtubules in several cell lines. Human FSD1 is associated with acetylated microtubule subsets, and the expression of FSD1 protects microtubules from depolymerization. FSD1 with N-terminal deletion binds to microtubules, but changes their structure. FSD1 separates from microtubules at the beginning of mitosis and recombines with microtubules at the beginning of cytokinesis. The ectopic expression of FSD1 inhibits the division and cytoplasmic division of Chinese hamster ovary cells. The gene encodes a Plekstrin homology and a protein containing the SEC7 domain, which acts as a guanine nucleotide exchange factor. The encoded protein regulates signal transduction by activating ADP-ribosylation factor 6 (provided by RefSeq, Aug 2012).

**MESP1** gene encodes members of the basic helix-loop-helix (bHLH) transcriptional regulatory protein family, which may play a role in the development of neonatal mesoderm (Saga et al. 1996). Lescroart et al. (2018) sequenced the single-cell RNA of wild-type and Mesp1-null cardiovascular progenitor cells (CPs) in mice. The results showed that the population of Mesp1 CPs was different in molecule and spanned the continuum of epiderm and late mesoderm cells (including hematopoietic progenitor cells).

**C7orf63** cilia and flagella-related proteins. In the olfactory epithelium, regulating the rate of activation and termination of odor response contributes to the stability of olfactory pathways (through similarity). Cilia are extensively involved in the development of organisms and various cellular functions. Little is known about how these genes contribute to neurodevelopmental disorders (Mahjoub 2013).

**References**

Atanasoski S, Toldo SS, Malipiero U, Schreiber E, Fries R, Fontana A (1995) Isolation of the human genomic brain-2/N-Oct 3 gene (POUF3) and assignment to chromosome 6q16. Genomics 26:272-280.

Belinson H, Nakatani J, Babineau BA, Birnbaum RY, Ellegood J, Bershteyn M, McEvilly RJ, Long JM, Willert K, Klein OD, Ahituv N, Lerch JP, Rosenfeld MG, Wynshaw-Boris A (2016) Prenatal β-catenin/Brn2/Tbr2 transcriptional cascade regulates adult social and stereotypic behaviors.Mol Psychiatry 21:1417-1433.

Berlin I, Schwartz H, Nash PD (2010) Regulation of epidermal growth factor receptor ubiquitination and trafficking by the USP8-STAM complex. J Biol Chem 285: 34909-34921.

Bill BR, Geschwind DH (2009) Genetic advances in autism: heterogeneity and convergence on shared pathways. Curr Opin Genet Dev 19:271-278.

Carim-Todd L, Escarceller M, Estivill X, Sumoy L (2001) Characterization of human FSD1, a novel brain specific gene on chromosome 19 with paralogy to 9q31. BiochimBiophysActa 1518:200-203.

Chen X, Yang Y, Liu J, Li B, Xu Y, Li C, Xu Q, Liu G, Chen YM, Ying J, Duan S (2017) NDRG4 hypermethylation is a potential biomarker for diagnosis and prognosis of gastric cancer in Chinese population. Oncotarget 8:8105-8119.

Choi GB, Yim YS, Wong HL, Kim S, Kim H, Kim SV, Hoeffer CA, Littman DR, Huh JR (2016) The maternal interleukin-17a pathway in mice promotes autism-like phenotypes in offspring. Science 351:933-939.

Clark AD, Oldenbroek M, Boyer TG (2015) Mediator kinase module and human tumorigenesis. Crit Rev Biochem Mol Biol 50:393-426.

de Leon-Bautista MP, Cardenas-Aguayo MD, Casique-Aguirre D,Almaraz-Salinas M, Parraguirre-Martinez S, Olivo-Diaz A, Thompson-Bonilla MD, Vargas M (2016) Immunological and Functional Characterization of RhoGDI3 and ItsMolecular Targets RhoG and RhoB in Human Pancreatic Cancerous and Normal Cells. PLoS ONE 11:e0166370.

Guilmatre A, Huguet G, Delorme R, Bourgeron T (2014) The emerging role of SHANK genes in neuropsychiatric disorders. Dev Neurobiol. 74:113-122.

Hashizume K, Yamanaka M, Ueda S (2018) POU3F2 participates in cognitive function and adult hippocampal neurogenesis via mammalian‐characteristic amino acid repeats. Genes Brain Behav. 17:118-125.

Hirosawa M, Nagase T, Ishikawa K, Kikuno R, Nomura N, Ohara O (1999) Characterization of cDNA clones selected by the GeneMark analysis from size-fractionated cDNA libraries from human brain. DNA Res 6: 329-336.

Hu VW (2012) Is retinoic acid-related orphan receptor-alpha (RORA) a target for gene-environment interactions contributing to autism?. Neurotoxicology 33:1434-1435.

Jaegle M, Ghazvini M, Mandemakers W, Piirsoo M, Driegen S, Levavasseur F, Raghoenath S, Grosveld F, Meijer D (2003) The POU proteins Brn-2 and Oct-6 share important functions in Schwann cell development. Genes Dev 17:1380-1391.

Kasher PR, Schertz KE, Thomas M, Jackson A, Annunziata S, Ballesta-Martinez MJ, Campeau PM, Clayton PE, Eaton JL, Granata T, Guillen-Navarro E, Hernando C, Laverriere CE, Lieden A, Villa-Marcos O, McEntagart M, Nordgren A, Pantaleoni C, Pebrel-Richard C, Sarret C, Sciacca FL, Wright R, Kerr B, Glasgow E and Banka S (2016) Small 6q16.1 Deletions Encompassing POU3F2 Cause Susceptibility to Obesity and Variable Developmental elay with Intellectual Disability. Am J Hum Genet 98:363-372.

Kerrisk CM, Sheng M (2018) USP8 deubiquitinates SHANK3 to control synapse density and SHANK3 activity-dependent protein levels. J Neurosci. 38:5289-5301.

Lei L, Chen MX, Chen S, Ao lJ (2018) Advances in research on the relationship between serotonin and autism. Shandong Medical Journal58:108-110. (Chinese)

Lescroart F, Wang X, Lin X, Swedlund B, Gargouri S, Sanchez-Danes A, Moignard V, Dubois C, Paulissen C, Kinston S, Gottgens B, Blanpain C (2018) Defining the earliest step of cardiovascular lineage segregation by single-cell RNA-seq. Science 359:1177-1181.

Lin Y J, Hsin I L, Sun H S, Lin S, Lai YL, Chen HY, Chen TY, Chen YP, Shen YT, Wu HM (2018) NTF3 is a novel target gene of the transcription factor POU3F2 and is required for neuronal differentiation. Mol Neurobiol. 55:8403-8413.

Mahjoub MR (2013) The importance of a single primary cilium. Organogenesis 9:61-69.

Manabe R, Whitmore L, Weiss JM, Horwitz AR (2002) Identification of a novel microtubule-associated protein that regulates microtubule organization and cytokinesis by using a GFP-screening strategy. Curr Biol 12: 1946-1951.

Piñero J, Queralt-Rosinach N, Bravo À, Deu-Pons J, Bauer-Mehren A, Baron M, Sanz F, Furlong L (2015) DisGeNET: a discovery platform for the dynamical exploration of human diseases and their genes. Database (Oxford) 2015:bav028.

Qu X, Jing L, Baldwin H S (2016) Postnatal lethality and abnormal development of foregut and spleen in Ndrg4 mutant mice. Biochem Biophys Res Commun 470:613-619.

Saga Y, Hata N, Kobayashi S, Magnuson T, Seldin MF, Taketo MM (1996) MesP1: a novel basic helix-loop-helix protein expressed in the nascent mesodermal cells during mouse gastrulation. Development 122:2769-2778.

Sarachana T, Zhou R, Chen G, Manji HK, Hu VW (2010) Investigation of post-transcriptional gene regulatory networks associated with autism spectrum disorders by microRNA expression profiling of lymphoblastoid cell lines. Genome Med 2:1-18.

Sato S, Tomomori-Sato C, Parmely TJ, Florens L, Zybailov B, Swanson SK, Banks CAS, Jin J, Cai Y, Washburn, MP, Conaway JW, Conaway RC (2004) A set of consensus mammalian mediator subunits identified by multidimensional protein identification technology. Mol Cell 14:685-691.

Schreiber E, Tobler A, Malipiero U, Schaffner W, Fontana A (1993) cDNA cloning of human N-Oct 3, a nervous-system specific POU domain transcription factor binding to the octamer DNA motif. Nucleic Acids Res 21:253-258.

Takaoka A, Wang Z, Choi MK, Yanai H, Negishi H, Ban T, Lu Y, Miyagishi M, Kodama T, Honda K, Ohba Y, Taniguchi T (2007) DAI (DLM-1/ZBP1) is a cytosolic DNA sensor and an activator of innate immune response. Nature 448:501-505.

Toma C, Hervás A, Balmaña N, Salgado M, Maristany M, Vilella E, et al (2013) Neurotransmitter systems and neurotrophic factors in autism: association study of 37 genes suggests involvement of DDC. World J Biol Psychiatry. 14:516-527.

Wang Z, Ma B, Li H, Xiao X, Zhou W, Liu F, Zhang B, Zhu M, Yang Q, Zeng Y, Sun Y, Sun S, Wang Y, Zhang Y, Weng H, Chen L, Ye M, An X, Liu J (2016) Protein 4.1N acts as a potential tumor suppressor linking PP1 toJNK-c-Jun pathway regulation in NSCLC. Oncotarget 7:509-523.

Warrier V, Baron-Cohen S, Chakrabarti B (2013) Genetic variation in GABRB3 is associated with Asperger syndrome and multiple endophenotypes relevant to autism. Mol Autism4:48.

Yamada Y, Nezu J, Shimane M, Hirata Y (1997) Molecular cloning of a novel vascular endothelial growth factor, VEGF-D. Genomics 42:483-488.

Yamamoto H, Kokame K, Okuda T, Nakajo Y, Yanamoto H (2011) Miyata, T. NDRG4 protein-deficient mice exhibit spatial learning deficits and vulnerabilities to cerebral ischemia. J Biol Chem 286: 26158-26165.

Yin AL (2012) Correlation between Ras/Raf/Erk1/2 signaling pathway overexpression and autism.Dissertation, Southern Medical University. (Chinese)

Zhang Z, She J, Yang J, Bu X, Ji G, Zhu S, He S, Chu D (2018) NDRG4 in gastric cancer determines tumor cell proliferation and clinical outcome. Mol Carcinog 57:762-771.
